# The Internet and HIV study: design and methods

**DOI:** 10.1186/1471-2458-4-39

**Published:** 2004-09-01

**Authors:** Jonathan Elford, Graham Bolding, Mark Davis, Lorraine Sherr, Graham Hart

**Affiliations:** 1 institute of Health Sciences & St Bartholomew School of Nursing and Midwifery, City University London, 24 Chiswell Street, London EC1Y 4TY; 2 Department of Primary Care and Population Sciences, Royal Free and University College Medical School, Rowland Hill Street, London NW3 2PF; 3 MRC Social and Public Health Sciences Unit, 4 Lilybank Gardens, Glasgow G12 8RZ

## Abstract

**Background:**

The Internet provides a new meeting ground, especially for gay men, that did not exist in the early 1990s. Several studies have found increased levels of high risk sexual behaviour and sexually transmissible infections (STI) among gay men who seek sex on the Internet, although the underlying processes are not fully understood. Research funded by the UK Medical Research Council (2002–2004) provided the opportunity to consider whether the Internet represents a new sexual risk environment for gay and bisexual men living in London.

**Methods:**

The objectives of the Internet and HIV study are to: (i) measure the extent to which gay men living in London seek sexual partners on the Internet; (ii) compare the characteristics of London gay men who do and do not seek sex on the Internet; (iii) examine whether sex with Internet-partners is less safe than with other sexual partners; (iv) compare use of the Internet with other venues where men meet sexual partners; (v) establish whether gay men use the Internet to actively seek partners for unprotected anal intercourse; (vi) determine the potential for using the Internet for HIV prevention. These objectives have been explored using quantitative and qualitative research methods in four samples of London gay men recruited and interviewed both online and offline. The four samples were: (i) gay men recruited through Internet chat rooms and profiles; (ii) HIV positive gay men attending an NHS hospital outpatients clinic; (iii) gay men seeking an HIV test in an NHS HIV testing or sexual health clinic; (iv) gay men recruited in the community.

**Results:**

Quantitative data were collected by means of confidential, anonymous self-administered questionnaires (n>4000) completed on-line by the Internet sample. Qualitative data were collected by means of one-to-one interviews (n = 128) conducted either face-to-face or on-line.

**Conclusion:**

The strength of the Internet and HIV study is its methodological plurality, drawing on both qualitative and quantitative research among online and offline samples, as well as taking advantage of recent advances in web survey design. The study's findings will help us better understand the role of the Internet in relation to gay men's sexual practice

## Background

Several studies have found elevated levels of high risk sexual behaviour among people who seek – and meet – sexual partners through the Internet [1-7] In a study conducted in a public HIV testing clinic in Denver, Colorado, for example, people who sought sex on the Internet were more likely to have had a sexually transmitted infection (STI) or report sexual exposure to a person with HIV than those who did not seek sex on the Internet [2]. They were also more likely to be male, gay and to report anal sex. While the study concluded that gay men were more likely than other participants to use the Internet to seek – and meet – sexual partners, it could not establish whether the excess sexual risk actually occurred with partners whom the men had met through the Internet.

A San Francisco study also found that gay men were more likely than heterosexual men and women to use the Internet to meet sexual partners [5]. Around one-third of the gay men interviewed at a public STI clinic had used the Internet to meet a sexual partner compared with one-in-ten heterosexual men and women. The gay men in the study said that their online partners – men they met through the Internet – were more likely to be casual (ie a one night stand) than their offline partners – men they met elsewhere.

Similar reports have also emerged from European studies. An investigation of gay men in London gyms found that in the year 2000 over one-third of those with Internet access had used it to look for sex [1]. When surveyed three years later, this figure had increased to nearly half [8]. HIV positive men were more likely to use the Internet to look for sex than HIV negative or never-tested men. Seeking sex on the Internet was associated with a recent STI diagnosis and high-risk sexual behaviour, ie unprotected anal intercourse (UAI) with a person of unknown or discordant HIV status [1]. This presents a risk for HIV transmission. In addition, HIV-positive Internet-sex seekers were more likely to report UAI with another HIV-positive man than those who did not seek sex in this way. This raises the possibility that HIV positive men use the Internet to meet other positive men for unprotected anal intercourse. While this does not present a risk of HIV transmission to an uninfected person, it may lead to co-infection with an STI or with another, potentially drug-resistant strain of HIV [9]. As in the U.S. studies, the London study could not establish whether the excess risk for HIV and STD occurred with sexual partners whom the men had actually met through the Internet.

The association between seeking sex on the Internet and high risk sexual behaviour raises a number of important, as yet unanswered questions. Does the excess risk for HIV and STI occur with sexual partners whom men actually meet through the Internet? Does the association reflect the fact that high risk men are selectively using the Internet to look for sex? Or does the Internet in some way facilitate high risk behaviour? Is the Internet attracting a constituency of men who have little contact with the established gay scene or health promotion agencies [10]? For example, men who do not use bars and clubs or bisexual men? In other words, does the Internet represent an emerging sexual risk environment for gay men? If so, what are the underlying processes? Answers to these questions are essential if we are to use the Internet effectively for HIV prevention and sexual health promotion.

To address these questions we have undertaken research funded for two years (2002–2004) by the UK Medical Research Council and the Department of Health. This has been conducted by researchers at City University London in collaboration with colleagues at the MRC Social and Public Health Sciences Unit Glasgow and University College London (see appendix 1).

### Research question

The core research question is: Does the Internet represent a new sexual risk environment for gay/bisexual men living in London?" (referred to as "gay men" throughout the rest of this paper). And if so, what are the underlying processes? The research focuses on gay men living in London since the incidence and prevalence of HIV infection among gay men in London is higher than elsewhere in the UK [11-13].

#### The objectives of the research are to

• measure the extent to which London gay men seek sexual partners on the Internet

• compare the characteristics of gay men who do and do not seek sex on the Internet

• examine whether sex with Internet-partners is less safe than with other sexual partners

• compare use of the Internet with other venues such as saunas and backrooms

• establish whether gay men use the Internet to actively seek partners for unprotected anal intercourse

• determine the potential for using the Internet for HIV prevention

## Methods

These objectives have been explored using both quantitative and qualitative research methods. While quantitative research methods can provide data on a range of outcomes they can rarely offer insight into underlying processes. Qualitative research, on the other hand, illuminates our understanding of human behaviour but is limited in the extent to which findings can be generalized. By adopting methodological plurality and employing both quantitative and qualitative methods, we will be able to build on the strengths of both approaches within an integrated research programme

In desribing the design and methods of the Internet and HIV study we focus on sampling strategy, recruitment into the study, data collection and data analysis.

### Sampling

Research among hard-to-reach groups such as gay men is usually based on convenience rather than probability samples [14]. For example, behavioural research among gay men in the UK has primarily been conducted among men recruited in bars, clubs, GUM clinics [15], gay pride events [16] and gyms [17]. While probability sampling would undoubtedly provide a more robust foundation for statistical analysis [18] such an approach is extremely, if not prohibitively expensive. Convenience samples have the advantage of being affordable and also provide the opportunity to focus on men with characteristics which may be of particular interest, eg men who report high risk behaviour [14]. The disadvantage of course is that such samples may introduce selection bias. This bias can be partially overcome by including samples from more than one source allowing for triangulation of data.

Consequently, we recruited gay men from four different sources – one online, three offline. Each sample has specific features which are pertinent to the research question.

#### HIV positive gay men

HIV positive men are central to any research concerning HIV risk, transmission and prevention [19-21]. Furthermore, HIV positive men are more likely to use the Internet to seek sexual partners than other men [1,8]. Consequently it was decided to over-sample HIV positive men for the study to permit an in-depth examination of sexual risk behaviour and use of the Internet in this group of men. In the UK everyone who is diagnosed HIV positive is offered free treatment and care within the National Health Service (NHS) through a hospital outpatients clinic. Consequently an NHS clinic sample will be broadly representative of all those living with diagnosed HIV.

#### Gay men seeking an HIV test

Most people seeking an HIV test have been at risk of HIV infection. Furthermore, HIV negative gay men with a history of multiple repeat testing report elevated levels of high risk sexual behaviour [22]. This group therefore merits inclusion in an investigation of the Internet as an emerging sexual risk environment. In the UK, the NHS offers free voluntary counselling and testing for HIV either in dedicated HIV testing clinics or in general sexual health clinics. Since there is no charge for this service, these clinics attract a broad cross section of people.

#### Gay men in the community

Surveying gay men in the community allows us to examine the extent to which they use the Internet for seeking sexual partners and the associated risks. Previous research has shown that gay men surveyed in central London gyms are broadly representative of men "on the scene" in London ie men who go to gay bars, clubs and other venues [23]. However, whereas questionnaires distributed in London bars and clubs have to be short because of the limited time available for completion, we have found that in gym-based surveys respondents are willing to complete questionnaires that take up to 15 minutes to answer. This allows for a detailed investigation of sexual behaviour.

#### The Internet

Men who use Internet chat rooms and profiles to seek sex with other men are clearly of central importance to this research project. The Internet may attract a constituency of men who would not otherwise be included in behavioural surveys among gay men ie men who do not go to gay bars, clubs or other venues and men who have not been tested for HIV [10]. Comparing the characteristics of men recruited online with the community and clinic samples will throw these differences, where they exist, into sharp focus.

### Recruitment

Based on previous research conducted among gay men in both clinic and community settings [1,8,22,23] we estimated that, for the quantitative arm of the study, we would needed to recruit 400–500 men in each of the four samples. This would provide sufficient power at a 5% level of significance to compare the characteristics of men who do and do not seek sex on the Internet, to compare use of the Internet with other venues such as saunas and backrooms, and to examine whether sex with Internet-partners is less safe than with other partners.

For the qualitative arm of the study, we recruited at least 20 men from each of the four samples to allow us to derive accounts of Internet dating and sexual practice from a diverse range of men, selected purposively according to age, education, employment, HIV status and use of the Internet for seeking sex.

#### HIV positive gay men

Men diagnosed with HIV infection attending an outpatient treatments clinic at the Royal Free Hampstead NHS Trust hospital, London over an 8 month period (October 2002–May 2003) were invited to participate in the research. Patients with a limited command of English were ineligible for the study as were those who were too ill to complete a questionnaire. Eligible patients were approached in the clinic's waiting area by a trained member of the research team who discussed the project with them. Patients were provided with written information about the research, contact details of the research team as well as helpline numbers. Once they had provided written consent, respondents were asked to complete a pen-and-paper questionnaire in the clinic and return it in a sealed envelope to the team member (further information about the questionnaire in Research Methods below). Some patients were only in the waiting area for a short time so there wasn't an opportunity to invite them to take part in the study.

Over the eight month survey period (October 2002–May 2003), 1001 individual male patients attended the clinic of whom 939 were deemed eligible for the study. Of those who were eligible, 864 were asked to complete a questionnaire and 620 did so. The response rate was 72% of men who were offered a questionnaire and 66% of all eligible men who attended the clinic. Of the 620 men who completed a questionnaire, 542 described their sexual orientation as gay or bisexual or had had sex with another man in the previous year. Of these, 523 men provided sufficient information to be included in the quantitative sample (table 1).

**Table 1:** Number of London gay/bisexual men who participated in the Internet and HIV study 2002–2003

	Quantitative arm					Qualitative arm				
		
Recruitment site	HIV positive	HIV negative	Never-tested	*Total*		HIV positive	HIV negative	Never-tested	*Total*
HIV treatment clinic	523	-	-	*523*		20	-	-	*20*
HIV testing & sexual health clinics*	15**	389	-	*404*		-	20	-	*20*
Community 2002	138	592	184	*914*	}	1	18	4	*23*
Community 2003	88	361	94	*543*	}				
Internet*** 2002	142	680	396	*1218*	}	17	35	13	*65*
Internet*** 2003	67	315	197	*579*	}				

* An additional 435 heterosexual men and 450 heterosexual women completed a questionnaire in the HIV testing clinic for the quantitative arm

* * Fifteen men received an HIV positive diagnosis when they returned for their test result

* * * An additional 3279 gay/bisexual men living in the UK but outside London completed the questionnaire online in 2002, 1944 in 2003 for the quantitative arm

HIV positive gay men who completed a questionnaire were asked on the last page if they would be willing to have an in-depth face-to-face interview, one-to-one, with a qualitative researcher working on the project (MD) (further information about the one-to-one interviews in Research Methods below). If they agreed, the researcher contacted them to arrange a time for the interview. This could be in the researcher's office at City University London, in the hospital or at the respondent's home. In this way 20 HIV positive gay men were recruited for one-to-one interviews as part of the qualitative arm of the study (table 1).

#### Gay men seeking an HIV test

People seeking an HIV test at the same-day HIV testing clinic, Royal Free Hampstead NHS Trust Hospital over a 13 month period (October 2002–November 2003) were invited to take part in the study. Based on previous research in the HIV testing clinic, we estimated that this would generate a sample of approximately 500 gay men, 500 heterosexual men and 500 heterosexual women [22]. Although the focus of the study was gay men, data were also collected from heterosexual men and women to provide a valuable comparison.

Using a similar strategy to the outpatient HIV treatments clinic, everyone seeking an HIV test was asked to participate in the research. Those who agreed completed a detailed, self-administered pen-and-paper questionnaire, after providing written consent, while they were waiting for their pre-test counselling. People with limited command of English were deemed ineligible for the study as were those who were too ill, too young (under 18 years) or too anxious to complete a questionnaire. Those attending the testing clinic on more than one occasion during the survey period were asked to only complete the questionnaire once.

Over the 13 month survey period (October 2002–November 2003), 1889 individuals came to the Royal Free clinic for an HIV test of whom 1753 were eligible for the study. Of those who were eligible, 1640 were asked to complete a questionnaire and 1230 did so. The response rate was 75% of people who were offered a questionnaire and 70% of all eligible persons who attended the clinic. Of the 1230 people who completed a questionnaire, 345 described their sexual orientation as gay or bisexual, 435 as heterosexual male and 450 as heterosexual female; 334 gay/bisexual men provided sufficient information to be included in the quantitative sample.

Gay men who completed a questionnaire were asked if they would be willing to have an in-depth, face-to-face interview with the qualitative researcher (MD). If they agreed, the researcher contacted them to arrange a time for the interview as described above. In this way, 16 gay men seeking an HIV test at the Royal Free were recruited for one-to-one interviews for the qualitative arm. Heterosexual men and women who completed the questionnaire were not asked to have a one-to-one interview.

Previous research suggested that the majority of clinic attenders would test HIV negative while approximately 6% of gay men were expected to test HIV positive. It was considered inappropriate to ask men who had just been diagnosed HIV positive if they would be willing to have a face-to-face interview. Consequently, the qualitative sample from the HIV testing clinic solely comprised men who had tested negative. Anyone who volunteered for an interview who subsequently received a positive test result was not included in the qualitative sample.

After 6 months it became apparent that while the response rate at the Royal Free HIV testing clinic was high the number of gay men seeking a test was not as large as had been expected. We decided therefore to extend recruitment to men seeking an HIV test at a sexual health clinic specifically for gay men at Barts and the London NHS Trust hospital, London. All men attending this clinic are gay.

Using a similar strategy to the one developed at the Royal Free, everyone attending the gay men's sexual health clinic at Barts and the London hospital between June and November 2003 was invited to participate in the research. Those who agreed completed a self administered questionnaire while they were waiting in the clinic for their appointment. Although all men were asked to complete a questionnaire, only men seeking an HIV test were eligible for inclusion in the Internet and HIV quantitative sample.

Over the 6 month survey period at the Barts and the London sexual health clinic (June – November 2003), 211 gay men came for an appointment of whom 209 were eligible for the study. Of those who were eligible, 198 were asked to complete a questionnaire and 156 did so. The response rate was 79% of men who were offered a questionnaire and 75% of all eligible men attending the clinic.

Of the 156 gay men who completed a questionnaire at Barts and the London, 70 were seeking an HIV test and provided sufficient information to included in the quantitative sample for the study. These men were also asked if they were willing to have a one-to-one interview with the qualitative researcher. Four men were recruited in this way for the qualitative arm of the study.

Combining the data from the two clinics, 404 gay men seeking an HIV test were recruited for the quantitative arm of the study between October 2002–November 2003, of whom 20 agreed to have a one-to-one interview for the qualitative arm (table 1). In addition 450 heterosexual women and 435 heterosexual men also completed a questionnaire for the quantitative arm.

#### Gay men in the community

Previous research has shown that gyms in central London provide a suitable environment for undertaking detailed behavioural research among gay men at risk of HIV infection [1,8,17,24,25]. In both 2002 and 2003, all men using any one of 7 central London gyms during a one-week period between January-March were invited to take part in the study. All these gyms have a substantial gay male membership. One gym was exclusively gay whereas the others estimated that gay men comprised 40–90% of their male membership [26]. All men using the gyms during the survey period were asked to complete a self-administered pen-and-paper questionnaire after providing written consent. A filter question on sexual orientation distinguished gay or bisexual men from straight men. Only gay/bisexual men were requested to answer questions on the Internet and sex. Men could complete the questionnaire in the gym or at home. Respondents returned completed questionnaires to collection boxes in the gym or by post to the research team.

In the mixed gyms the number of questionnaires handed out to gay men was estimated by multiplying the total number of questionnaires distributed in the gym by the proportion of male members who were gay (according to the managers' estimates). To calculate the response rates we divided the number of questionnaires returned by gay men (as indicated on the questionnaire) by the estimated number of questionnaires handed out to gay men, as described above. In 2002, 921 gay men completed the questionnaire while in 2003, 550 men did so. Those men who provided information on their HIV status (2002, n = 914; 2003, n = 543) were included in the quantitative sample (table 1). The estimated response rate each year was 50%–60%.

In both years, gay men who completed a questionnaire were asked if they would be willing to have an in-depth, face-to-face interview with the qualitative researcher (MD). If they agreed, the researcher contacted them to arrange a time for the interview as described above. Some interviewees were recruited through snowballing. In this way, 23 men were recruited from the gyms for the qualitative arm of the study (table 1).

#### The Internet

In both 2002 and 2003, men using UK chat rooms or personal profiles on gaydar ()or gay.com () were invited to take part in the study. Gaydar and gay.com are two of the UK's most popular websites for gay men (personal communication H Badenhorst, M Watson). Over a four week period in May-June each year, a series of pop-ups and banners advertised the research project in UK chatrooms and on profiles pages. Clicking on a popup or banner took men to the homepage of the online questionnaire. Men who agreed to complete the questionnaire then did so after providing informed consent – all online.

For technical reasons, it was not possible to restrict banner advertising or pop-ups to London chatrooms or personal profiles alone. Instead, the advertising was restricted to *UK *chatrooms or profiles. Consequently, anyone entering a UK chatroom or profile during the survey period had the opportunity of completing the online questionnaire even though the target group was London gay men.

In 2002, 1250 London men completed the online questionnaire while in 2003, 595 did so. Those men who provided information on their HIV status were included in the quantitative sample (2002, n = 1218; 2003, n = 579) (table 1). A further 3279 men living in the UK but outside London completed the questionnaire in 2002; 1944 in 2003. The decline in the number of respondents in 2003 compared with 2002 reflects a general pattern seen by gaydar and gay.com in other online surveys in the UK (personal communication H Badenhurst, M Watson).

Estimating a response rate for the online quantitative sample is problematic [27,28]. It is impossible to gauge what proportion of chatroom and profile users saw the banners and pop-ups advertising the online survey. Nor do we know what percentage of those seeing the pop-ups and banners went on to complete the questionnaire. Based on estimates provided by  and  on the number of people using their Internet chatrooms and profiles during the survey periods, it is likely that less than one percent of all users completed the questionnaire. This level of response is standard for online surveys. This highlights the importance of not relying solely on respondents recruited through the Internet for research of this kind.

Gay men living in London who completed the online questionnaire were asked if they would be willing to have a one-to-one interview with the qualitative researcher (MD). Those who agreed were asked to send an email to the researcher who then contacted them, also by email, to provide further information about the study and to arrange a time for the interview as described above. Interviews were either conducted online or face-to-face (see Research methods below). Of the London men who completed a questionnaire online in 2002 or 2003, 65 went on to have a one-to-one interview as part of the qualitative arm (table 1); 30 men were interviewed face-to-face while 35 were interviewed online

### Data collection

#### Quantitative data

The questionnaires sought detailed information on the men's socio-demographic characteristics (age, ethnicity, employment, education), sexual orientation, HIV test history (date and result of last test), history of STIs, access to and use of the Internet, seeking sex on the Internet, use of other venues (eg back rooms, saunas), as well as sexual risk behaviour in the previous 3 months, differentiating between Internet- and other sex partners. Unprotected anal intercourse (UAI) in the previous 3 months was categorized according to type (regular or casual) and HIV status of partner. Detailed information was collected about sexual behaviour with partners met through the Internet and with partners met elsewhere in order to compare the level and nature of risk with Internet and other sexual partners. Data were also collected on potential confounding factors such as recreational drug use, alcohol consumption, relationships, attitudes towards new treatments for HIV and mental health. Standard and validated questionnaire items were used extensively (copies of the questionnaires are available from JE).

Core questions, included in the questionnaires for all four samples, were worded identically to ensure direct comparability between the different groups. In addition, some questions specific to each group were included eg, detailed questions on HIV test history and reasons for testing for people seeking an HIV test; questions on HIV medication, CD4 and viral load for HV positive men. The questionnaires were piloted both online and offline among gay men at the developmental stage of the study and revised in the light of any feedback and comments.

People recruited offline, in clinics or gyms, completed a pen-and-paper questionnaire. All questionnaires were confidential and anonymous. They contained no information that would allow an individual respondent to be identified. For the HIV positive men, information on their most recent viral load and CD4 count was abstracted from hospital records and linked to their questionnaire without breaching confidentiality. The HIV test result of those seeking an HIV test was linked to their questionnaire, again without breaching confidentiality. Once the questionnaire and clinic data had been linked in the database individual identifiers (eg hospital numbers) were removed and destroyed to ensure anonymity.

Men recruited through Internet chatrooms and profiles completed the questionnaire online. The online questionnaire was constructed by a computer programmer at gaydar () working in close collaboration with the research team. Because of their technical expertise and capacity, gaydar hosted the questionnaire on their server. All questionnaires were confidential and anonymous. Identifiers such as IP addresses were removed from the questionnaires completed online before the data were downloaded to a database.

Within each sample men were asked (a) to complete only one questionnaire and (b) whether they had been in any of the other samples. For example, men who completed the questionnaire online were asked whether they had also completed a questionnaire in the gyms or clinics, etc.

#### Qualitative data

Qualitative data were collected by means of one-to-one, in-depth interviews conducted face-to-face (n = 93) or on-line (n = 35)

##### Face-to-face interviews

The interviewees were volunteers from the quantitative samples recruited in the HIV treatments clinic, HIV testing clinic, gyms or online as described above. They provided written consent for the one-to-one interview which lasted between 50 and 90 minutes and was audio-taped for transcription. The interviews were generally conducted in the research office at City University London but occasionally at the interviewee's home or in the clinic. Confidentiality was provided in two ways; first, the interviewee's contact details were not linked to the interview transcript and secondly personal identifiers such as the person's name, where he was born, lived or worked were removed from the transcripts. Once all the data had been transcribed and entered into the database, individual identifiers (eg interviewees' contact details) were destroyed.

##### Online interviews

The interviewees were volunteers from the *online *quantitative samples. They provided informed consent by email. Interviews were prearranged and conducted in a private room on  or  which only the interviewer and interviewee could enter. The interviews were synchronous and conducted entirely through text generated as online chat. Each interview lasted between 50 and 90 minutes at the end of which the interview text was copied and pasted into a Word document. Confidentiality was provided in the same manner as the face-to-face interviews..

Interviews were conducted in two phases. Phase one (n = 24 interviews) focused on how gay men used the Internet for sexual partnering while phase two (n = 104) focused on risk behaviour related to the Internet.

The interviews for *phase one *were based on a topic guide comprising questions about age, residence, schooling, employment, relationship status and HIV testing. Social and sexual lifestyles were explored in depth paying particular attention to the role of the Internet. The topic guide included seeking sexual partners, preferences for different ways of meeting partners (including the Internet), Internet experience and skills, learning how to use the Internet for sexual partnering, online communication skills, other uses of the Internet and related media, a recent sexual episode and its relationship with the Internet.

The topic guide for phase two comprised social background; sexual lifestyle and role of the Internet; risk episodes including a description of anal sex with a condom and without a condom with Internet and non-Internet partners; HIV testing (where relevant); general discussion of Internet experience and web profiles; safer sex (rules, negotiated safety, serostatus of sexual partner, disclosure, slip-ups, problems, pleasure and rationality); and sources of knowledge and skills about risk reduction (health carers, media, school, common sense). For HIV positive men the topic guide also covered their experience of HIV treatment and care (eg current diagnosis, treatments, treatment effectiveness, clinical markers) and the relationship between treatments and risk (eg prospects for new drugs, role of viral load in risk taking, reinfection);

#### Ethics

The research protocol was approved by the following ethics committees: Royal Free Hospital and Medical School Local Research Ethics Committee, the East London and The City Research Ethics Committee and City University London Research Ethics Committee.

### Sample characteristics and data analysis

Over 4000 London gay men were recruited for the quantitative arm of the study in 2002 and 2003 from the HIV treatments and testing clinics, gyms and through the Internet. Of these, a subset of 128 men were interviewed one-to-one for the qualitative arm (table 1)

#### Quantitative data

Data from the pen-and-paper questionnaires were coded, entered into a database and verified. Data collected online were downloaded directly into a database. The background characteristics of the different samples in the quantitative arm are presented in table 2.

**Table 2:** Background characteristics of the men in the quantitative sample, by recruitment site

	HIV treatment clinic		HIV testing clinics		Community				Internet			
				
	2002–2003 (n = 523)		2002–2003 (n = 404)		2002 (n = 914)		2003 (n = 543)		2002 (n = 1218)		2003 (n = 579)	
	n	%	n	%	n	%	n	%	n	%	n	%
Age (median; range)	38	*23–70*	32	*17–73*	35	*17–79*	36	*18–75*	33	*18–70*	32	*18–75*
Ethnicity (white)	467	*89.6*	342	*84.7*	821	*90.4*	484	*89.6*	1117	*91.7*	526	*89.3*
Employed	324	*62.8*	317	*91.4*	771	*85.3*	450	*83.2*	1007	*82.7*	477	*80.2*
Higher education	328	*65.7*	261	*72.9*	761	*83.8*	425	*79.7*	810	*66.5*	377	*63.9*

Sexual orientation "gay"	489	*93.5*	354	*87.6*	869	*95.1*	523	*96.3*	1084	*89.0*	524	*88.1*
In a relationship with a man	285	*55.3*	223	*55.9*	469	*51.6*	300	*55.4*	531	*43.6*	254	*42.7*
HIV positive	523	*100.0*	15	*3.7*	138	*15.1*	88	*16.2*	142	*11.7*	67	*11.3*

Treatments optimism 1	201	*40.9*	92	*26.1*	175	*20.3*	120	*23.3*	164	*14.5*	79	*14.2*
Treatments optimism 2	141	*28.7*	85	*24.6*	176	*20.7*	117	*22.9*	158	*15.5*	94	*17.0*
Uses recreational drugs	277	*60.6*	200	*60.4*	467	*53.2*	334	*64.4*	497	*40.8*	212	*38.4*

Felt depressed	274	*55.2*	85	*49.6*	389	*44.5*	253	*48.6*	552	*45.3*	274	*48.7*
Had suicidal thoughts	96	*21.9*	46	*14.3*	104	*12.8*	65	*12.8*	210	*17.3*	105	*18.7*

Has access to the Internet	443	*86.3*	367	*90.8*	841	*92.6*	499	*93.1*	1193	*97.9*	588	*98.8*
Uses the Internet to seek sex	223	*43.6*	186	*46.0*	400	*44.4*	280	*52.0*	1040	*85.4*	544	*91.4*
Has sex with men only	453	*96.2	352	*87.6*	853	*93.5*	509	*93.7*	1089	*89.4*	537	*90.3*

STI in previous 12 months	13	*27.5*	79	*19.8*	203	*22.5*	127	*23.5*	295	*24.2*	128	*21.7*
Non-concordant UAI	116	*22.2*	141	*34.9*	199	*22.1*	119	*22.1*	391	*32.1*	196	*32.9*
Concordant UAI only	58	*11.1*	36	*8.9*	135	*15.0*	80	*14.8*	175	*14.4*	87	*14.6*

Completed clinic survey ^1^	-	-	-	-	-	-	16	*3.0*	-	-	26	*4.7*
Completed gym survey ^2^	25	*5.0*	14	*3.9*	-	-	-	-	24	*2.0*	16	*2.9*
Completed 2002 online survey ^3^	27	*5.4*	26	*7.3*	-	-	54	*10.2*	-	-	-	-

Treatments optimism 1: Men who agreed with the statement "I am less worried about HIV now that treatments have improved"

Treatments optimism 2: Men who agreed with the statement "I believe new treatments make people with HIV less infectious"

Non-concordant UAI: unprotected anal intercourse in the previous 3 months with someone of unknown or discordant status Concordant UAI only: unprotected anal intercourse in the previous 3 months only with someone of the same HIV status.

* as a percentage of sexually active men (some men had not had sex in the previous year)

^1 ^Number (%) of men completing a 2003 questionnaire in the gyms or online who said they had also completed a questionnaire in the HIV testing clinic or treatments clinic

^2 ^Number (%) of men completing a questionnaire online or in the clinics who said they'd also completed a questionnaire in a gym

^3 ^Number (%) of men completing a questionnaire in the gyms (2003) or in the clinics who said they'd also completed the 2002 online questionnaire

In all samples, the majority of men were relatively young, white and identified as gay. There were however, differences between the samples in these and other characteristics. For example, men who completed a questionnaire online were less likely to describe themselves as gay than men who completed a questionnaire in a gym (88% v 96% in 2003). On the other hand, men surveyed online were more likely to have used the Internet to look for sex (approximately 90%) than men who surveyed in the clinics or gyms (40–50%). The differences between the samples will be explored further in future analyses and subject to formal statistical testing. For the most part the four samples were independent; only a minority of men said they had completed a questionnaire in more than one recruitment site (eg in a clinic and in a gym); range 2–10%.

Data analysis, using standard statistical packages, will allow us to examine; whether sex with Internet partners is of higher risk than with other men (within-person analysis) and the extent to which Internet sex seekers were specifically looking for unprotected anal intercourse either online or offline. The characteristics of those who have and have not used the Internet to find a sexual partner will be compared to explore whether selected groups of men (eg bisexual or those at high risk) seek sex on the Internet. The importance of the Internet in relation to other venues will be examined. The samples, ranging in size from 404 to 1218, are sufficiently large to allow us to detect statistically significant differences between and within the different groups at the 5% level of significance.

All analyses will be conducted for HIV positive, negative and never-tested men separately and comparisons will be made within and between the four samples. Comparison of those recruited through the Internet with the community and clinic samples will indicate whether the Internet attracts men who would otherwise be hard to reach for health promotion and HIV prevention. The data collected online from men living in the UK but outside London will provide opportunities for further analysis. For example their characteristics and behaviours can be compared with those of the London men surveyed online as well as with other men living outside London *surveyed in the community *(eg in Scotland). The data collected online will also allow us to examine patterns of Internet use in a sample.that covers the whole of the UK.

A PhD studentship, funded separately by the UK Economic and Social Research Council (ESRC), will allow us to examine methodological issues around using the Internet for data collection and research [29], such as mode effect, motivation for participating in online surveys, number of fields completed online and offline, drop outs and non-probability sampling (see appendix 2). The ESRC-funded PhD will utilize all the data collected from men living in the UK who completed an online questionnaire in 2002 or 2003.

In addition, data collected in the HIV testing clinic will also allow us to explore Internet sex-seeking and risk behaviours among heterosexual men and women.

#### Qualitative data

The interview transcripts from both the face-to-face and online interviews were coded and analysed using Nvivo. The background characteristics of the men interviewed for the qualitative arm of the study are summarized in table 3.

**Table 3:** Background characteristics of the men in the qualitative sample, by recruitment site

	HIV treatment clinic		HIV testing clinics		Community		Internet			
				
Interviewed.......	Face-to-face (n = 20)		Face-to-face (n = 20)		Face-to-face (n = 23)		Online (n = 35)		Face-to-face (n = 30)	
	n		n		n		n		n	
Age (median; range)	38	*31–59*	40	*25–66*	35	*24–51*	32	*20–63*	39	*21–60*
Employed	9		18		18		30		23	
Higher education	15		15		17		20		20	
Has regular sexual partner	8		8		12		14		12	
HIV status										
*HIV positive*	20		-		1		6		11	
*HIV negative*	-		20		18		20		15	
*Never-tested*	-		-		4		9		4	
Seeks sex through the Internet										
*Rarely, never or used to*	13		14		11		6		0	
*Seeks sex both on and offline*	3		4		5		10		14	
*Seeks sex mostly through the Internet*	4		2		7		19		16	

Interviewees were recruited for qualitative interviews once they had completed a behavioural questionnaire for the quantitative arm of the study. Additional purposive criteria were adopted to ensure the sample included men from a range of age groups; of different HIV status (HIV positive, HIV negative and never tested); of varying educational attainment; both employed and unemployed; and who reported differential use of the Internet for sexual purposes. Online, as well as face-to-face (FTF) interviews were conducted to encourage the participation of gay men who used the Internet to look for sex. This combination of matching the quantitative arm in terms of recruitment sites, together with additional purposive criteria and two interviewing methods resulted in 128 qualitative interviews with a diverse range of gay/bisexual men. All interviewees lived in the greater London area.

For the qualitative arm of the study, we addressed analytic bias using guidance provided by Barbour [30], Kvale [31] and Popay & Williams [32] for whom methodological rigour is best addressed through the process of qualitative research. For example, the internal logic of the study; emphasis on the quality of the craft of research together with communicative and pragmatic forms of validation [31]; "privileging subjective meaning" [32]. In our study we addressed quality in three main ways:

• Iteration

• Team analysis

• Transparency

##### Iteration

We conducted a mapping phase to provide an empirical context for our thinking about the Internet-related sexuality of gay men and to orient further research in terms of the sampling and topic guides. We also adopted the practice of constant comparison derived from grounded theory as another form of iteration.

##### Team analysis

The research team was involved in a cycle of reflection on the data as they were generated and analysed. In the first meeting the team reviewed a transcript to identify possible themes for analysis. Notes and the transcription of the meeting contributed to the formulation of themes. At an intermediate stage the team was asked to apply the thematic framework to other transcripts. This helped to assess the utility of the framework and elaborate possible new themes. A second meeting was then held where the team reviewed the coding scheme for the qualitative research and its application to the entire data set. This process was conducted twice – first for data generated during phase 1, then for data collected in phase 2.

##### Transparency

We documented how the data were generated, catalogued and analysed to make it open to observation.

The one-to-one interviews will be analysed for recurring and contradictory themes relating to the core research questions. Phase one will help orientate the qualitative research around the Internet and risk. Phase two will provide information about the range of sexual experiences linked to the Internet as well as how people communicate about sex and risk through the Internet. The qualitative interviews will allow for greater interrogation than is permitted by self-completed questionnaire alone. A number of issues will be examined including personal experiences of using the Internet for sex, partner selection, the context in which unsafe sex occurs, the emergence of social and sexual networks via the Internet and the value or meaning placed on unsafe sex with casual partners met in different settings including the Internet.

## Discussion

Does the Internet represent a new sexual risk environment for gay men? To answer this question, we have employed a range of quantitative and qualitative research methods in online and offline samples of London gay men. The data we collect will allow us to explore in depth the association between seeking sex on the Internet and high risk behaviour and also consider the underlying processes.

One of the strengths of the Internet and HIV study is its methodological plurality. Detailed behavioural data have been collected in the quantitative arm of the study from a large number of men recruited in community and clinic settings as well as through the Internet. Questionnaires were completed online by the Internet sample. On the other hand, qualitative interviews will allow for a greater understanding of the context in which unsafe sex occurs and the processes that underlie the behavioural patterns seen in the quantitative analysis. By using both quantitative and qualitative methods, we hope to garner the best that both approaches can offer within an integrated research programme

Sampling men online has allowed us to develop innovative research methods for both the quantitative and qualitative arms of the study by taking advantage of recent advances in web-based data collection. The online questionnaire provided opportunities for innovation with respect to its design, format of the questions and data entry. Conducting one-to-one interviews online has opened up new ways of undertaking qualitative research. Ours is one of a small number of studies which have explored this approach to qualitative interviewing [33]. In addition, the ESRC-funded PhD examining the Internet and research methodology will provide an opportunity to consider the advantages and disadvantages of conducting research online [29].

The Internet undoubtedly offers enormous potential for HIV prevention and sexual health promotion [34,35]. Our research will reveal whether the Internet reaches a group of men who have little contact with the established gay scene or health promotion agencies. If this is the case, the Internet could provide access to an otherwise hard-to-reach group of men. Exciting as these new opportunities are, however, we still know relatively little about the efficacy of online sexual health promotion and HIV prevention [36]. Data generated by both the quantitative and qualitative arms of our study will provide a better understanding of the social and sexual networks created through the Internet. Who is using the Internet to look for sex and how are they using it? In this way the potential for using the Internet for sexual health promotion and HIV prevention can be established

## Competing interests

None declared.

## Authors' contribution

JE, GH and LS conceived the study; all authors participated in its design; JE was responsible for overall project management; GB was responsible for conducting the quantitative arm of study; MD was responsible for the qualitative arm; JE drafted the manuscript with input from GB and MD. All authors read, revised and approved the final manuscript.

## Appendix 1

*The Internet and HIV study: MRC-funded research*

City University London, Institute of Health Sciences, St Bartholomew School of Nursing and Midwifery

Professor Jonathan Elford, principal investigator

Graham Bolding, research fellow (quantitative arm)

Mark Davis, research fellow (qualitative arm)

MRC Social and Public Health Sciences Research Unit, Glasgow

Professor Graham Hart, co-investigator

Royal Free and University College Medical School, London

Professor Lorraine Sherr, co-investigator

Collaborators

, , Royal Free Hampstead NHS Trust Hospital London, Barts and the London NHS Trust Hospital, Camden Primary Care Trust, London, Central London gyms.

The research was funded from 2002–2004 by the UK Medical Research Council and the Department of Health as part of its AIDS Epidemiological Research Programme (G0100159. The Internet and HIV: an examination of high risk sexual behaviour among London gay men who seek sex on the Internet)

Further information about the project can be found on our website at 

## Appendix 2

*The Internet and research methodology: ESRC-funded PhD studentship*

City University London, School of Social Sciences and Institute of Health Sciences, St Bartholomew School of Nursing and Midwifery

Alison Evans PhD candidate

School of Social Sciences and Institute of Health Sciences, St Bartholomew School of Nursing and Midwifery

Professor Dick Wiggins first supervisor,

School of Social Sciences, Department of Sociology

Professor Jonathan Elford second supervisor,

Institute of Health Sciences, St Bartholomew School of Nursing and Midwifery

*Advisory Board*

City University London

Graham Bolding, Mark Davis, Professor Jonathan Elford, Professor Roger Jowell, Professor Dick Wiggins

MRC Social and Public Health Sciences Research Unit, Glasgow

Professor Graham Hart

Royal Free and University College Medical School, London

Professor Lorraine Sherr

Further information can be found at 

## Pre-publication history

The pre-publication history for this paper can be accessed here:


